# Celebrating 125 years of the synapse: from Sherrington to the present day

**DOI:** 10.1042/NS20220015

**Published:** 2022-12-22

**Authors:** S Clare Stanford

**Affiliations:** Department of Neuroscience, Physiology and Pharmacology University College London, London, U.K.

**Keywords:** Behaviour, neural circuits, New technologies, Sherrington, Synapse

## Abstract

This themed collection celebrates 125 years of the synapse through a series of reviews written by a team of international experts in the field. The first in the series explains Sherrington’s contribution to the debate about the term ‘synapse’ and its function in neuronal signaling. The topics that follow cover recent developments in a wide range of topics: new technologies for research of synaptic structure; proteomics and the regulation of synaptic integrity and function; their role in the processing of information in thalamic neuronal circuits; and how genetic mutations can modify synaptic function in ways that can have profound effects on mood, cognition and behaviour.

This themed collection celebrates two anniversaries linked to Charles Sherrington. It is 125 years since he added the term, ‘synapse’, to the scientific vernacular and 90 years since he was awarded the Nobel prize for his work on spinal reflexes, for which the role of synapses is fundamental.

The collection opens with an article from the acclaimed biomedical historian, Tilli Tansey, who charts the early history of research in this field [1]. She starts with the days when the synapse was little more than a theoretical concept, fostered by histological evidence from Ramón y Cajal, and outlines Sherrington’s tentative thoughts on what to call it. This is followed by a fascinating record of the debate about whether or not synaptic communication between neurons was mediated directly, by electrical coupling, or indirectly, via a chemical messenger (‘spark or soup’). The article outlines each of the important contributions to the field from Sherrington and other pioneering physiologists, including Edgar Adrian, John Langley, Thomas Elliott, Henry Dale and Otto Loewi, amongst others, and concludes with the evidence that finally convinced John Eccles that chemical neurotransmission prevailed. The present article sets the scene for the whole collection and adds perspective to landmark achievements that are discussed in this collection.

In the next article of the series, Avila and Henstridge take a giant leap forward from Cajal’s work and describe awesome developments in techniques for imaging synapses [2]. The baseline for this technology was the refinement of electron microscopy (and the development of epoxy resins), which enabled the high resolution, 2D visualization of biological tissues, including neurons and their synapses. The article explains how it is now feasible to use block-face trimming which, in combination with sophisticated data handling, enables the assembly of 3D constructs of synapses and the mapping of their connections within the brain matrix. Not only that, but this technology can be combined with more traditional histological techniques, such as immunolabelling, to map the distribution of specific proteins within the same 3D construct. So far, this technology is not widely accessible, but is surely an exciting sign of what can be achieved in the future.

The pros and cons of other techniques, such as immunoelectron microscopy, confocal microscopy and mass spectrometry-based proteomics, are covered, but a major focus of this article is array tomography (AT), particularly immunofluorescent AT. This technique enables serial immunoflourescence of individual AT sections using standard epifluorescence microscopy and can be used to produce 3D stacks of tissue samples. The authors offer helpful advice on how to use this technique, including how to access the necessary software. They go on to describe examples of how AT is being used to study postmortem brain tissue from humans and rodents to gain insight into synaptic biology (the connectome) and the pathology of neurodegenerative diseases. Finally, they discuss how AT can be combined with other imaging techniques, including conventional electron microscopy, to gain even more information about the 3D structure of, and protein expression in, synapses.

A major change in our understanding of synapses since Sherrington’s day is that we now know that they are not fixed entities, but are constantly being dismantled and reassembled as an essential component of neurotransmission, synaptic stability and plasticity. The next article, by Marijn Kuijpers [3], reviews the latest developments in our understanding of the multiple pathways that contribute to regulation of the proteosome, which are essential for the architectural integrity and function of synapses. The article starts with the role of ubiquitin, ubiquitin-ligases and deubiquitating enzymes and how they contribute to the regulation of presynaptic protein turnover (proteostasis) and how they deal with neuronal wear and tear, and repair. The endolysosomal pathway and autophagy, as crucial elements of maintaining synaptic viability, are covered next. The role of proteostasis in learning and memory, natural ageing and neurodegenerative disorders are all highlighted, together with important questions in the field that should be a priority for future research.

The collection then moves on from studies of synapses, specifically, to explore their role in neuronal signaling in complex neuronal networks. In a fascinating review, Copeland and Salt [4] discuss the role of the thalamus in processing of afferent information of all modalities and, in particular, the pivotal contributions of Group II (metabatropic) mGlu receptors. The article starts with an explanation of the complex circuitry within and between the thalamic nuclei, and their connections with up- and down-stream brain regions. All aspects of the field are covered, including the mapping of the distribution of this group of receptors within the thalamic circuitry, their pharmacology, orthosteric ligand selectivity and the influence of allosteric modulators, electrophysiological properties and fluorescence imaging *in vitro*. All the evidence, gathered from these diverse technologies, are appraised and assembled to deduce the role of Group II mGlu receptors in neuronal signaling at different key points in the thalamus. The review concludes with a discussion of the contribution of these receptors to information processing and cognitive performance, more generally, as well as how their dysfunction could help explain prevalent human disorders, such as chronic pain, schizophrenia and epilepsy.

Finally, Pohl and Hörnburg discuss postsynaptic elements of the regulation of synaptic signaling. Importantly, these authors also address the question of the extent to which is it likely that genetic alterations can produce animal models that recapitulate full-blown human disorders versus the possibility that they account for changes in specific elements of a disorder, be they physiological and/or behavioural aspects of the phenotype (i.e., domains or endophenotypes). The strong heritability of neurodevelopmental disorders make these ideal candidates for addressing such questions and this review discusses research of autism to illustrate how they are being investigated. The article focuses on a group of genes for cell adhesion molecules, specifically the neuroligins [5], which govern the formation of glutamatergic (excitatory) and/or GABAergic (inhibitory) synapses; mutations in the genes encoding these molecules are implicated in several neurodevelopmental disorders, including autism.

The authors acknowledge the difficulty of studying the effects on behaviour of combinations of genetic mutations (and their interaction with environmental factors) and so have focused on mutations with pronounced effects on the function of the expressed neuroligins and what is known about the underlying neurobiology in different brain regions. As the authors point out, such research can help to identify convergence of the effects of a range of genetic mutations on molecular pathways, or even specific proteins, on changes in the behavioural phenotype, which could also help to decode individual differences in behaviour.

Despite covering a wide range of research of synapses (from their structure, protein composition, functional regulation, role in neurocircuitry and even their influence on behaviour), this collection gives a mere glimpse of the progress that has been made since Sherrington's day. He would surely be amazed at what has been achieved.

## Data Availability

There are no data to share.

## Open Access

Open access for this article was enabled by the participation of University College London in an all-inclusive *Read & Publish* agreement with Portland Press and the Biochemical Society under a transformative agreement with JISC.

## Abbreviation

AT: array tomography
